# Anionic and Ampholytic High-Amylose Starch Derivatives as Excipients for Pharmaceutical and Biopharmaceutical Applications: Structure-Properties Correlations

**DOI:** 10.3390/pharmaceutics15030834

**Published:** 2023-03-03

**Authors:** Marc-André Labelle, Pompilia Ispas-Szabo, Salma Tajer, Yong Xiao, Benoît Barbeau, Mircea Alexandru Mateescu

**Affiliations:** 1 Department of Chemistry, Research Chair on Enteric Dysfunctions ‘Allerdys’, CERMO-FC Center, Université du Québec à Montréal, C.P. 8888, Succursale Centre-Ville, Montréal, QC 3PC 3P8, Canada; 2 Department of Biological Sciences & CERMO-FC Center, Université du Québec à Montréal, C.P. 8888, Succursale Centre-Ville, Montréal, QC H3C 3P8, Canada

**Keywords:** pH sensitive biopolymers, hydrogels, self-assembly, pharmaceutical excipients, drug delivery systems, functional films, structural analysis

## Abstract

Many chemical modifications of starch are realized in organic (mostly methanol) phase, allowing high degrees of substitution (DS). Some of these materials are used as disintegrants. To expand the usage of starch derivative biopolymers as drug delivery system, various starch derivatives obtained in aqueous phase were evaluated with the aim to identify materials and procedures which would generate multifunctional excipients providing gastro-protection for controlled drug delivery. Chemical, structural and thermal characteristics of anionic and ampholytic High Amylose Starch (HAS) derivatives under powder (P), tablet (T) and film (F) forms were evaluated by X-ray Diffraction (XRD), Fourier Transformed Infrared (FTIR) and thermogravimetric analysis (TGA) methods and correlated with the behavior of tablets and films in simulated gastric and intestinal media. At low DS, the HAS carboxymethylation (CMHAS) in aqueous phase, generated tablets and films that were insoluble at ambient conditions. The CMHAS filmogenic solutions, with a lower viscosity, were easier to cast and gave smooth films without the use of plasticizer. Correlations were found between structural parameters and the properties of starch excipients. Compared to other starch modification procedures, the aqueous modification of HAS generated tunable multifunctional excipients that may be recommended for tablets and functional coatings for colon-targeted formulations.

## 1. Introduction

Starch is a biopolymer of interest due to its capacity of self-assembly [1]. low cost and biocompatibility, in addition to its susceptibility to be hydrolysed by salivary and intestinal α-amylase. Various sources of starch and numerous physical, chemical or enzymatical modifications were proposed to extend its range of applications [2]. 

Regarding the starch origin, the ratio between the two components (amylose and amylopectin) present in the starch granule will define many of its properties [3]. Certain soluble starch powders are largely used as tablet disintegrants, binders or fillers. Differently, the high amylose starch (HAS) powder, less popular because it is amorphous, has low solubility and, it is able however to form strong matrices and modulate the drug release [4]. Also, compared to other starches, the HAS has shown slower hydrolysis by α-amylase (of either pancreatic or colonic bacteria origin) and is therefore suitable as an excipient for colonic delivery [5]. 

Between chemical modifications that can be operated on the polysaccharidic chains of starch, the cross-linking and the grafting of various functional groups are frequent. Cross-linked high amylose starch (CLHAS-CL) devices loaded with ciprofloxacin have been proposed as an option for long-lasting implants in the treatment of osteomyelitis [6]. Transarterial embolization is a low-invasive treatment of solid tumors. Cross-linked starch microspheres slowly-biodegradable by serum α-amylase, known as Degradable Starch Microspheres (DSM) or Spherex^®^ have been described as candidates for clinical translation [7]. Other new applications include resorbable stents with shape memory [8], bone scaffolds [9,10], wound dressing materials [11], tumor targeting [12] and molecular probes [13]. For bone tissue engineering, the viability and proliferation of cultured cells were remarkably increased with polyhydroxybutyrate (PHB)-starch scaffolds compared to PHB control samples [14]. 

Grafting of anionic groups (carboxymethyl CM) on starch is usually performed in organic phase to obtain high yields and elevated degree of substitution (DS) [15,16,17,18,19,20]. Sodium starch glycolate is a commercial carboxymethyl potato starch with superdisintegrant properties [21]. Contrarily, when the carboxymethylation of HAS is done in aqueous phase it generates excipient materials with different properties, allowing a tunable drug release delay. The swelling and drug release rates from CMHAS tablets based on powders obtained via an aqueous procedure may be adjusted by varying the DS, the protonation ratio of the CM groups and the drying procedure. All parameters have a tailoring role in the micro-organization of starch chains [22,23]. Anionic carboxymethyl starch can be used as pH-responsive pharmaceutical excipient. The aqueous carboxymethylation of HAS with low DS produced a semi-amorphous starch excipient. When exposed to gastric acidity, the tablets based on CMHAS will form an outer compact layer due to protonation of carboxylic groups preventing thus the acidic medium to penetrate the tablet [24]. In the intestinal fluid (at neutral pH), the outer CM groups are deprotonated, ionized, and hydrated. The tablets swell, and by erosion or disintegration, release the drug. These structural changes related to pH were valorized in formulation of gastro-resistant tablets for oral delivery of various drugs including bioactive agents such as pancreatic enzymes [25], F4 fimbriae oral vaccine, probiotics (*Lactobacillus rhamnosus*) [26,27,28]. Allowing a tunable release delay, CMHAS was also described as an excipient for drug chronodelivery [29]. The process of aqueous carboxymethylation, being ‘solvent-free’ and of low-cost, is of interest for industrial purposes and for “green” pharmaceutical products.

Starch may also be used under film form with pharmaceutical applications i.e., oral fast-disintegrating films [30,31], transdermal delivery [32] and soluble coatings for tablets. There are many reports on films based on common starches (i.e., corn, potato, rice) with plasticizers, but only a few described plasticizer-free films made of modified HAS [33]. Acetylated HAS is film-forming material but in the context of colon-targeting tablet coatings, it was not possible to use it alone, the addition of ethyl cellulose being required to enhance its solubility [34,35,36]. 

In this study, different excipients were obtained by aqueous-phase modifications of HAS and it was interesting to investigate their matrix-forming and filmogenic properties. The report is focused on the physical-chemical characterization of different starch derivatives and on the correlation of their properties under powder, tablet and film forms. A more complete understanding of such structural modifications will be useful to expand the range of applicability of starch-based materials in the pharmaceutical field. Such correlations can also be important tools for formulators in selection of type of starch and modification level, depending on specific application.

## 2. Materials and Methods

### 2.1. Reagents

Hylon VII^®^ (~70% amylose), Melojel^®^ (~28% amylose) and PenPure60^®^ native potato starch were generous gifts from Ingredion (Westchester, IL, USA). Potato amylose (100%) and potato amylopectin (100%) were purchased from BDH Biochemicals (Chemical Division, Toronto, CA, USA). Vivastar^®^ was a gift from JRS Pharma (Patterson, NY, USA). Chloroethylamine hydrochloride (CEA), glycidyl trimethylammonium chloride (GTMAC), sodium carboxymethyl cellulose (CMC) with a MW of 90,000 g/mol, sodium monochloroacetate (SMCA), sodium trimetaphosphate (STMP) and other chemicals were all reagent grade and used as received from Millipore Sigma (Burlington, MA, USA). Pancreatin from porcine pancreas (8× concentrated) was purchased from Millipore Sigma. The proposed time to simulate gastric transit is 2 h in simulated gastric (SGF) followed by simulated intestinal fluid (SIF) The SGF was made with 7 mL HCl (37% *w*/*w*) and 2 g NaCl for 1 L (as described by the USP-43, NF 38 [37]). The SIF was prepared with 6.8 g monobasic potassium phosphate in 750 mL distilled water and 77 mL 0.2 N NaOH and adjusted to pH 6.8 with 0.2 N NaOH as described by the USP-43, NF 38 [37].

### 2.2. Starch Modifications

The procedures and the derivatives are presented in Figure S1 and summarized in Table 1. The following parameters were examined: (i) amylose/amylopectin ratio (by selection of different starch sources), (ii) gelatinization conditions (temperature, medium, time) and (iii) chemical modifications (crosslinking, grafting of ionic groups). 

Starch carboxymethylation (CMHAS#1) (Figure S1) was done as previously described [25]. Briefly, 140 g HAS were suspended in 340 mL water at 55 °C with a benchtop stirrer. Then, 320 mL of 2M NaOH were added. After 20 min of gelatinization, 70 g of sodium monochloroacetate (SMCA) were added under stirring. The mixture was kept under agitation for 1 h, cooled, neutralized with acetic acid, and precipitated with aqueous methanol (60% *v*/*v*). The precipitate was washed with aqueous methanol (60% *v*/*v*) until the conductivity was below 50 μS and finally dried with acetone and filtered. 

CMHAS#2 was obtained by heating the HAS suspension at 90 °C for 1 h before adding NaOH and proceeding with carboxymethylation as for CMHAS#1 except that the amount of SMCA directly in powder form was doubled. CMHAS#3 was synthesized as the CMHAS#1 but with a gelatinization time extended to 1 h.

HAS was cross-linked (HAS-CL20) using the same gelatinization parameters as for CMHAS by adding sodium trimetaphosphate (STMP) at 20 g/100 g of starch.

CMHAS was also cross-linked to obtain CMHAS-CL10. The crosslinking was repeated with the slurry method using only half the volume of water, thereby obtaining the hereto called CMHAS-CL10#2. Corn (CS) and potato starch (PS) were carboxymethylated and cross-linked to generate CMCS-CL10 and CMPS-CL10, for comparison. Two different ampholytic derivatives (carrying both aminoethyl and CM-groups) were also synthesized (Figure S1). The starch hydroxyls were substituted with quaternary ammonium functions by reacting with glycidyltrimethylammonium chloride (GTMAC) during the synthesis of CMHAS, as previously described [38], leading to the formation of (2-hydroxypropyl) trimethylammoniumcarboxymethylHAS (TMACMHAS). TMACMHAS cross-linked with STMP was also prepared, in a one-step reaction, termed TMACMHAS-CL10. Lastly, HAS was aminated with 2-chloroethylamine hydrochloride (CEA) and carboxymethylated, generating AECMHAS, as previously reported [39].

**Table 1:** Synthesis in aqueous phase of starch derivatives.

Type of Modification	Physical	Chemical (g Reagent/100 g Starch)			Derivative Name	DS_titr_	Ref.
Type of Starch	Gelatinization Step	Cross-Linked with STMP	Grafting Ionic Groups				
			Anionic with SMCA	Cationic			
HAS	20 min aqueous 2M NaOH	20 g	-	-	HAS-CL20	0.00	[40]
		-	50 g	-	CMHAS#1	0.15	[25]
	60 min H_2_O, boiled	-	100 g	-	CMHAS#2	0.34	-
	60 min aqueous 2M NaOH	-	50 g	-	CMHAS#3	0.19	-
		10 g	75 g	GTMAC 97.6 mL	TMACMHAS-CL10	0.35	[38]
		-	75 g	CEA 75 g	AECMHAS	0.22	[18]
	20 min aqueous 2M NaOH	10 g	50 g	-	CMHAS-CL10 #2	0.19	[41]
Corn			50 g	-	CMCS	0.15	[25]
Potato		10 g	50 g	-	CMPS-CL10	0.22	[40]

### 2.3. Characterization of Powders

The degree of substitution (DS) of the CM-starch is defined as the number of substituted hydroxyls on each glucose unit (maximum 3). Determination of the DS by back titration was done as per literature [42]. A sample is protonated in 2 M HCl for 30 min, filtered and washed as previously detailed. The final pH of wet powders after full protonation was around 3.2. The FTIR band at 1593 cm^−1^ corresponding to carboxylate group was absent, triggering the appearance of the band at 1650 cm^−1^ attributed to the carboxylic acid group. For each sample, approximatively 100 mg were accurately weighed and dissolved in 0.05 M NaOH (with little heating if needed) and then titrated with 20 mL of 0.05 M HCl using phenolphthalein as indicator. The DS was calculated from Equation (1), where 162 g/mol is the molecular weight of a glucose unit, 58 g/mol is the increase in molecular weight accounted for each CM group substituted and m_dry_ is the mass of the dry sample.

DS = 162 × n_COOH_/ m_dry_ − 58 × n_COOH_ (1)

The amount of CM groups (n_COOH_) is given by Equation (2), where V_b_ is the volume of HCl used for the titration of the blank, V is the volume of titration of the sample and C_HCl_ is the concentration of the HCl.

n_COOH_ = (V_b_ − V)C_HCl_ (2)

X-ray diffraction (XRD): Diffractograms were recorded on a Bruker D8 (Billerica, MA, USA), from 5 to 35° (2θ). Powders were flattened in the sample holders, tablets were fitted with the height of the holder and the films were fixed to a glass square with tape on the sides. Baselines were fitted with 8 points and the relative crystallinity (RC) was calculated as the percentage of peak area from the total area under the curve (by the software). A new parameter, the percentage of V-type (%V), was calculated as the intensity of V-type peaks (13.4 and 19.8 °2θ) from the total intensity of the cumulative peaks.

Fourier-transformed infrared (FTIR) spectroscopy was used to evaluate the short-range order in starch structures. The C-O-H bending bands 1044, 1035, 1022 and 995 cm^−1^ may be used to estimate changes in ordered and amorphous starch chains (Figure S2). The amount of crystallinity (AOC), related to the gelatinization/retrogradation process, was evaluated by the ratio 1044/1035 [43]. The ratio 995/1022 represents the crystallinity index, CI, defined as the smectic organization of the helices [44]. The ratios were compared after two different correction methods and in relation to different deconvolution parameters (Tables S1 and S2).

Viscosity was evaluated for each starch material in 2% (*w*/*v*) aqueous solutions at 25 °C ± 2 °C (using an oscillating viscometer SV-10 (A&D Ltd., Japan), with a small sample adaptor for 40 mL. Vivastar^®^ is very hydrophilic and consequently its viscosity (higher than that of HAS and of CS) was measured at 1% (*w*/*v*).

Thermogravimetric analysis. Approximately 3 mg of each sample were analyzed without further conditioning. The thermogravimetric curves were recorded on a TGA Q500 (TA Instruments, New Castle, DE, USA) from 25 to 375 °C under nitrogen conditions. The degradation onset was determined by intersection of the slope line with the linear part of the curve. The water content was estimated by the weight loss measured at 150 °C (end of the slope). The temperature for 50% weight loss (T_w50_) was previously reported to be representative of the amylose content of starch samples [45]. In this study, the change of slope for HAS materials was interfering with the measurement of T_w50_. For this reason, T_w50_ was also compared with T_w60_ (the temperature at 60% weight loss), and to the main peaks of the differential thermogravimetric curves (DTG_max_). 

The blue value (BV) is a parameter related to the capacity of starch to form helical complex with iodine. It was used to estimate the impact of the modifications on the structure. The BV test was done as previously reported [46]. Absorbances of the starch-iodine complex were analyzed in triplicate between 200 and 900 nm with a Libra S50 spectrophotometer (Biochrom US, Holliston, MA, USA) using a quartz cuvette. The BV was reported as the absorbance of the samples at 640 nm (A_640_). The apparent amylose content (AAC) was calculated from a linear regression using pure amylose and pure amylopectin [47]. The ratio A_525_/A_640_, representative of the amylose:amylopectin ratio [48,49], was also analyzed. 

### 2.4. Tablet Preparation and Testing

The starch powders (300 mg) were compressed in a 9.54 mm diameter cylindrical mold with a manual hydraulic press (Carver, Inc., Savannah, GA, USA) at 2 tons for 10 s. The powders were conditioned at ambient environment without further treatment prior to compression. Tablet hardness was evaluated on a BenchSaver™ Series hardness tester (Varian, Inc., Cary, NC, USA). Structural parameters were evaluated by XRD and FTIR, as described in Section 2.3.

Tablet water uptake—The tablets (300 mg) were incubated in an incubator shaker at 37 °C, 100 rpm, 2 h in 40 mL SGF followed by 2 h in 40 mL SIF and weighed after each step. Results are expressed as the mass of the wet tablets.

Disintegration test—A disintegration bath (Electrolab disintegration tester EDi-2, Betatek Inc., Toronto, ON, Canada) was used following the recommendation of the United States Pharmacopeia (USP <701>) on physical testing (chapter 701-disintegration). The containers were first filled with simulated gastric fluid (SGF) for 2 h and then replaced by simulated intestinal fluids (SIF) for the remaining time. Initially, tablets were allowed to hydrate 4–5 min prior to placing the floating disks in the cylinders.

### 2.5. Film Casting and Testing

Preparation: the films were obtained in hexagonal polystyrene molds by casting (evaporation of the filmogenic compositions) in ambient conditions. The preparations were made with 3.5% *w*/*w* starch derivatives in distilled water, without plasticizers. For better homogenization, solutions were heated in a boiling water bath and then agitated slowly on a rotary agitator. After heating, the viscosity was lower, generating filmogenic formulations with an average density of 0.99 ± 0.02 g/mL. The hexagonal molds (diameter of 76 mm, area of 37.5 cm²) were filled with 14 g of each filmogenic preparation (0.37 g/mm²) and left to stand on a leveled surface at ambient conditions for 2 days. 

Testing of the films. The films were analyzed by TGA, XRD and FTIR methods, as previously described. 

Moisture absorption—Chambers were equilibrated with controlled relative humidity (RH) using different saturated salt solutions (200 mL) for at least one week. NaI, NaBr and Nal were used for 40, ~60 and 75% (*w*/*v*) RH, respectively. Films were kept in the chambers for at least 3 days and their water uptake was expressed as percentage of their change in weight. 

Water uptake—Films were immersed into SGF or SIF, delicately removed, carefully wiped, and weighed at specific time intervals for 1 h. The water uptake was expressed as the mass of the wet films. 

Solubility—Dry films were weighed, submersed into SGF or SIF for 2 h (with or without enzymes), removed and air-dried at 60 °C until a constant weight was reached (about 3 days). Solubility was calculated as the percentage of weight loss based on the initial film weight.

### 2.6. Cytotoxicity of Various Starch Derivatives

The HRT-18 is an epithelial cell line derived from a colorectal adenocarcinoma. Cells were maintained in alpha MEM (Minimum Essential Medium) supplemented with 10% fetal bovine serum and antibiotics (1% penicillin-streptomycin) in 5% CO_2_ at 37 °C. HRT-18 cells (3 × 10^5^) were treated with CMHAS, AECMHAS or Vivastar^®^, all at a final concentration of 0.05 mg/mL, in addition to H_2_O_2_ (positive control) and after two days, assayed for cell viability using XTT (2,3-bis (2-methoxy-4-nitro-5-sulfophenyl)-2H-tetrazolium-5-carboxanilide salt). The XTT tetrazolium assay is based on the reduction of XTT by mitochondrial NADH enzymes converting XTT to formazan crystal (Adan et al., 2016). Briefly, the medium was removed followed by addition of 125 µL XTT solution (XTT 1 mg/mL, 7.5 mg/mL PMS phenazine methosulfate) for a 3 h incubation. Absorbances were measured at 450 nm. Untreated cells (Mock) were set at the 100% viability value. 

## 3. Results 

It was of interest to correlate the structural data of the obtained starch derivatives in powder, tablet and film form (Table 2) to their behavior as potential multipurpose excipients for monolithic tablets or as coating films affording gastro-protection. The TGA parameters, crystallinity, blue value, density and viscosity, diminished when increasing the modification. This is due to a lower organization, smaller crystals, helices altered by the modifications and to an increased solubility. These results are detailed and discussed below.

### 3.1. Characterization of Powders

Under a powder form, derivatives were mainly evaluated in relation to the degree of organization of polysaccharidic chains in crystalline or amorphous regions. The X-ray diffractometry is a reference method in starch structural analysis, allowing to observe the crystallinity resulting from the organization in ordered layers and allows quantification of the relative crystallinity (% RC). In aqueous phase, the crystallinity of a starch excipient is related to its retrogradation. This reorganization phenomenon may induce twisting or cracking of the cast films. The crystallinity may also be related to the chemical modification: the new functional groups grafted on the starch chains will hinder the retrogradation/crystalline forms. 

**Figure 1:**
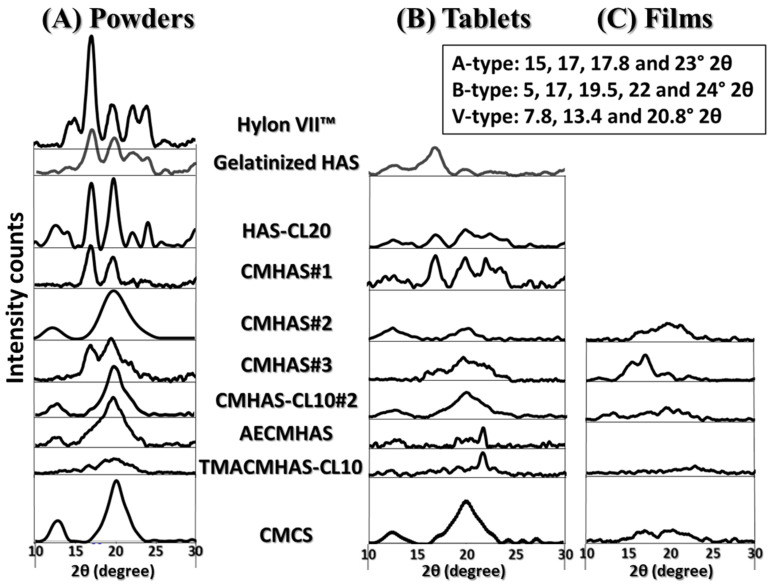
Diffractograms of the powders (**A**), tablets (**B**) and films (**C**) based on high-amylose starch (HAS) and derivatives and on CM corn starch (CMCS).

The ratio of type A or B peaks to the V-type peaks may be indicative of the amylose to amylopectin ratio in the large crystalline regions. The diffractograms are presented in Figure 1A and the calculated RC and percentage of V-type (%V) organization are presented in Table 2 as long-range organization parameters. Gelatinization alone had a low impact on the crystallinity. The CMHAS#1 was obtained using a short gelatinization time prior to modification, and the remaining crystallinity was higher than for other derivatives. Amylopectin-related morphology (B-type) was still present (17°), as much as for CMHAS#3 (long gelatinization time), but both had a low DS (0.17) and a single type of chemical modification. Nevertheless, all derivatives had lower crystallinity than the gelatinized HAS, meaning that the presence of carboxymethyl functions greatly hinders the retrogradation. The TMACMHAS-CL10 was the most amorphous material, due to the 3 types of chemical modifications plus 1 h gelatinization time.

For CMHAS-CL10, the slurry method with higher concentration of NaOH (Table 2) may have more drastically disorganized the starch, inducing irreversible changes. In addition to physical modifications due to gelatinization, the additional chemical modification may also have been more efficient on the amylopectin, preventing its reorganization. The absence of amylopectin-related peaks for CMCS shows that it is more easily disorganized.

In HAS, it was proposed that amylose could be organized in crystalline structures [50,51] and even participate in forming double helices [51,52]. A partial remaining crystallinity after modifications on HAS was already reported and ascribed to better matrix-forming properties [53]. 

Information available on evaluation of short-range organization parameter using FTIR to follow physical or chemical modification is scarce. The state (i.e., dry, hydrated, paste, film, tablet) of the tested samples renders analysis even more difficult. In the new prepared derivatives, after carboxymethylation of HAS, a new band was observed around 1590 cm^−1^ (Figure S2), attributed to new carboxylate functions. The spectra in region 800–1185 cm^−1^ (insert of Figure S2) were corrected, deconvoluted and normalized to analyze the bands 995, 1022, 1035 and 1044 cm^−1^. Different parameters of FTIR band ratios were investigated (data in supplementary materials Tables S1 and S2). CI values for the amorphous HAS derivatives were lower than those of the modified amorphous corn starch and of the modified potato starch (Table 2, Figure S3). CI may therefore be a better indicator of crystallinity for amylopectin than for amylose. The changes in CI ratios correlated well with the gelatinization extent. The parameters of log-range and short-range organization are reported in Table S2.

**Figure 2:**
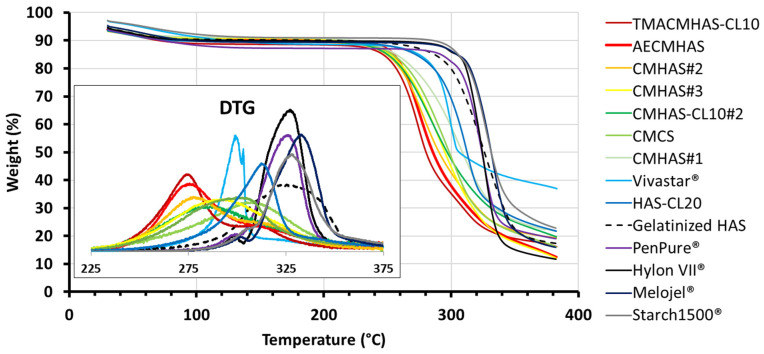
The TGA thermograms of the powder samples scanned at a heating rate of 10 °C/min. The insert shows the differential TGA (DTG).

Viscosity (Table 2) varies extensively depending on the physical and chemical modifications, but also on the macro-organization of the granules, while parameters from the TGA (onset, T_W60_, T_W50_ and DTG_MAX_) showed a decrease corresponding to the extent of the modifications (Figure 2): i.e., the DS or the presence of different functional groups (Table 2 and Table S3). Possibly, TGA data were related to the organization, whereas variation in viscosity was multifactorial (depending on macro-organization, DS, type of functional groups). Overall, the DTG_MAX_ was related to the crystallinity of samples, being lower for more amorphous and more hydrophilic excipients.

The blue value (BV) can give insights on the starch structure integrity. The absorbance spectra of the native starches showed a good correlation with the amylose:amylopectin ratio. The BV, the apparent amylose content (AAC) and A_525_/A_640_ ratio were reported for native and modified starches (Table 2) and details of the analyses are added in supplementary material (Table S4). Overall, the decreasing BV and AAC correlated with structural alteration due to gelatinization conditions and to steric hindrance caused by te chemical modifications. This resulted in a lower capacity to complex iodine and less coloration. Previous reports have mentioned that these values might depend on the degree of polymerization of amylose [54,55], the experiment setup [56] and the DS of modified starch [46].

### 3.2. Characterization of Tablets

Low density modified starches gave strong monolithic matrices. The water content of the starch influences the compression behavior (reorganization) and the cohesiveness (H-bonding) of the resulting materials [57], modulating their hardness (not reported) and swelling (Figure S4). When the starch was dried before compaction, tablet swelling was different. It was observed that drying of the powders improved their flow properties and density, but it also impacted hydration, which was rapid on the surface of the tablet. This may be due to the absence of water to plasticize the starch upon compaction, leading to fragile tablets with different hydration speed. Compression of dried starch likely gave more porous matrices. Without water as a binder, holes and imperfections in the matrix are more probable and would accelerate fluid penetration. 

The XRD profiles of starch tablets (Figure 1B) showed lower relative intensities and lower RC than the corresponding powders. The RC decreased after compaction (Table S2), which may be caused by compression of the granules and disturbance of the crystalline organization. A decrease in crystallinity is an usual observation for compaction [58], concomitantly to a broadening of the peaks due to crystal deformation [59]. For all samples, amylopectin- and amylose-related crystallinities were reduced after compression (Figure 1, Table S2). 

#### 3.2.1. Water Uptake of the Tablets

**Figure 3:**
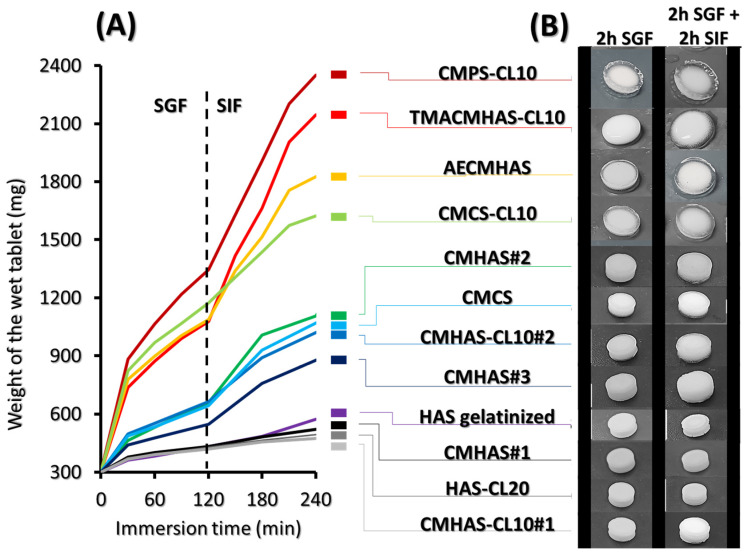
The weight of the tablets during water uptake (**A**) and pictures of tablets after 2 h in SGF, followed by a 2 h incubation in SIF) (**B**).

The weight of the tablets during immersion is reported in Figure 3. The formation of a gel layer on the tablet surface is the combination of different processes. The solubility of starch arises after physical and chemical modifications (i.e., gelatinization, grafting) or due to the type of drying process, all factors impacting the organization of polysaccharidic chains. Due to tablet gelation (becoming a gel after hydration), the erosion rate of the tablet needs to be slower than that of its hydration, particularly for sustained release purposes.

The powders with lower levels of organized polysaccharidic chains (resulting from the gelatinization step) generated strong tablets and denser granules, whereas aggregated powders gave friable tablets. Many modified HAS did not swell (gelatinized HAS, HAS-CL20, CMHAS#1, CMHAS-CL10#1), some swelled slightly (CMHAS#2, CMHAS#3, CMHAS-CL10#2 and CMCS) whereas others swelled greatly (CMPS and TMACMHAS derivatives without cross-linking). The CMPS had a high solubility due to the presence of potato starch. The solubility of TMACMHAS was related to the functional groups. Both were also obtained with cross-linking and tablets based on CMPS-CL10 or TMACMHAS-CL10 formed outer gel layers after immersion in SGF, showing that cross-linking created a stronger network. Furthermore, phosphate groups from the STMP can be easily protonated thereby contributing to the outer gel layer formation.

The CMCS-CL10 (based on corn starch) was gel-forming and its weight increased constantly in both media. The water uptake of ampholytic starches AECMHAS and TMACMHAS-CL10 was slower in SGF than in SIF due to the ionic stabilisation of ammonium with carboxylic groups. The weight gain was faster in SIF due to deprotonation of carboxylic functions. The higher solubility of ampholytic materials carrying ammonium salt groups resulted from protonation of amine in acidic medium.

#### 3.2.2. Erosion Rate of the Tablets

The drug-free starch tablets were evaluated for 2 h in SGF and than in SIF in the presence of pancreatic amylases (Table 3). When exposed to these media, the ampholytic TMACMHAS-CL10 tablets were disintegrated after 3 ½ h, followed by tablets based on CMHAS#2, the CMCS-CL10, CMPS-CL10 and CMHAS#3. Tablets of gelatinized HAS started eroding after 7 h. The HAS-CL20, CMHAS#1, CMHAS-CL10#1, CMHAS-CL10#2 and CMCS tablets were not affected after 9 h but showed different behaviors: the first three were only hydrated, whereas the latter two formed gels. This different erosion behaviour indicates that the modified HAS derivatives are tunable matrix-forming materials.

### 3.3. Characterization of the Films

#### 3.3.1. The Filmogenic Solutions and Aspect of Films

**Figure 4:**
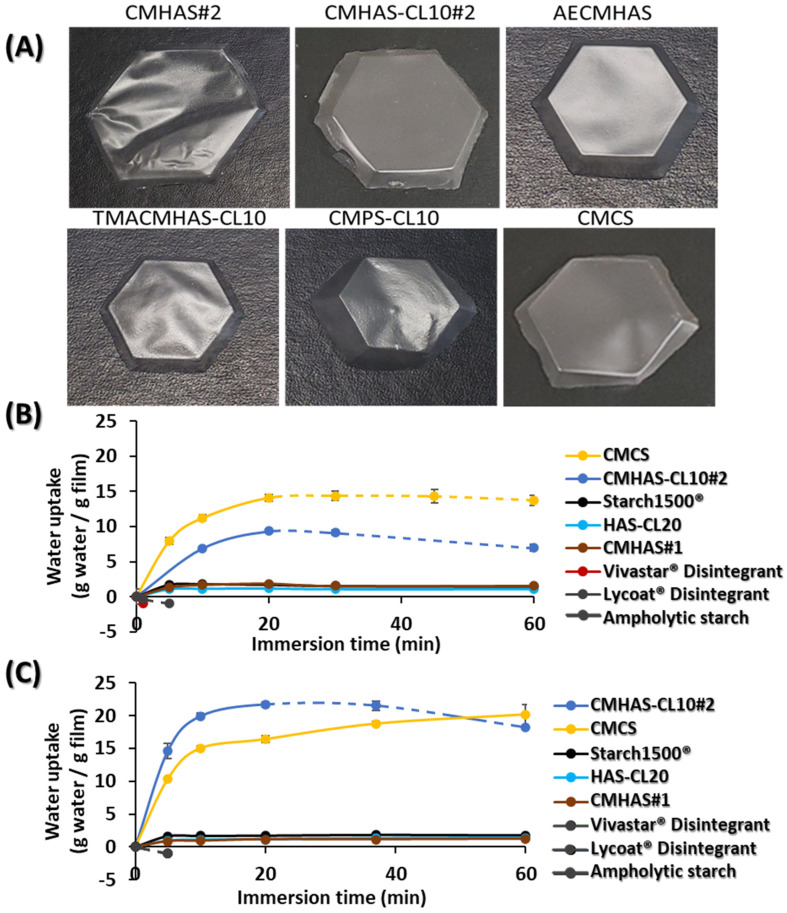
Pictures of the films obtained by casting of 3.5% (*w*/*v*) of corresponding aqueous solutions of HAS (CMHAS#2, CMHAS-CL10#2, ampholytic starches AECMHAS and TMACMHAS-CL10), potato (CMPS-CL10) and corn (CMCS) starch derivatives (**A**). Water uptake of the films after immersion in SGF (**B**) and in SIF (**C**); dot line: beginning of erosion.

By using amorphous powders, it was possible to obtain films with interesting visual aspects (Figure 4A). The amorphous materials (CMHAS#2, CMHAS#3, CMHAS-CL10#2, CMCS) generated flat, smooth, and flexible films, probably due to the chemical modifications that prevented retrogradation. The physical modification (gelatinization) of HAS in aqueous NaOH allows preparation of powders that are fully soluble in cold water. With a low amylopectin content, the modified HAS generated easy to handle low viscosity preparations (Table 4). The filmogenic solutions of cross-linked HAS-CL20 and CMHAS-CL10#2 presented a higher viscosity, and homogeneous spreading was limiting. The non-gelatinized Hylon VII^®^ suspension was not filmogenic, even after moderate heating due to its high amylose content generating strong H-bonds and crystallinity. CMHAS#1 and HAS-CL20 powders kept B-type and V-type crystallinity; they were not soluble and required heating to generate filmogenic solutions. The films were rigid and not completely flat when cast from solution without heating.

Water content of the films—Initial water content (at room condition), measured by TGA, was between 8% (*w*/*v*) and 9% (*w*/*v*).

Moisture absorption—After conditioning at controlled relative humidity (40, 60 and 75% RH), changes in weight of the films were noted (Table 4). A slight increase (from 40 to 60% RH) was measured. At 75% RH, all films presented higher water content. The ampholytic TMACMHAS-CL10 was very sticky and could not be weighed appropriately. Solubility of the corresponding powder derivatives was in fact related to the moisture absorption of the films.

#### 3.3.2. Structural Studies of the Films

The diffractograms of the films are presented in Figure 1C and related parameters are summarized in Table S2. CMHAS#2 had small peaks of V-type and B-type helices, while CMHAS-CL10#2 (slurry-type treatment) mostly contained V-type helix.

Band ratios (Table S2) were measured on the FTIR region 800–1185 cm^−1^. They were similar for HAS, potato, or corn starch films, suggesting that the organization after casting was similar, irrespective of the starch source or derivatives.

For the films, the maximal values of DTG increased in the following order: CMHAS#2 < TMACHAS-CL10 < AECMHAS < CMHAS#3 < CMHAS-CL10#2 < CMCS, showing a good correlation with their solubility (TGA and DTG parameters of the films are reported in Table S3). This suggests that the displacement of the DTG maxima may be linked to the amorphous character of the initial powders and that the amorphous powders may generate more amorphous films compared to other starches.

Effect of casting on structural organization was estimated in powders versus films. The short-range order (in FTIR) increased, while the long-range order (crystallinity by XRD) decreased (Figure 1) suggesting that films had smaller crystal size and that the amylopectin, with small chains, retrograded, whereas amylose did not. The amylose participates in the formation of a homogeneous network, in which the entanglement prevented retrogradation. It was previously suggested that amylose would reorganize around the amylopectin clusters and that the amylose:amylopectin ratio was an important parameter for the formation of a network [60]. Regular starch films were reported to retrograde extensively. However, in films from modified HAS, this was prevented by the formation of such network. The averaged TGA data show that the final weight, onset and DTG peak were higher for the films than for the powders (Tables S3 and S5).

#### 3.3.3. Behavior of the Films in Acidic (SGF) and Neutral (SIF) Media

Water uptake—The weight of the films after immersion in SGF were compared with their weight after immersion in SIF (Figure 4B). CMHAS-CL10#2 and CMCS were both hydrated forming thick gel sheets that resisted to immersion in SGF for 1 h. They had similar behavior in SIF but at this neutral pH, CMHAS-CL10#2 was more hydrated, showing a pH-dependent behavior, whereas the CMCS had the same water intake at both pH values.

Solubility of the films made of HAS-CL20, CMHAS#1 or CMHAS#3 remained unchanged in the enzyme-free SGF/SIF system (Table 4). CMHAS#2 films were soluble in both media due to higher DS (0.34). The AECMHAS films remain intact in SGF and were solubilized in SIF, also showing a pH-dependent behavior, whereas TMACMHAS-CL10 films were solubilized in both media possibly related to the presence of basic TMA groups. Overall, HAS derivatives had lower solubility than the other starches.

This shows that the films based on CMHAS with low DS obtained from aqueous synthesis would be stable at acidic pH during the gastric transit and would be mainly degraded in SIF in the presence of α-amylase.

#### 3.3.4. Factors Influencing the Properties of the Films from Different Starch Types

**Figure 5:**
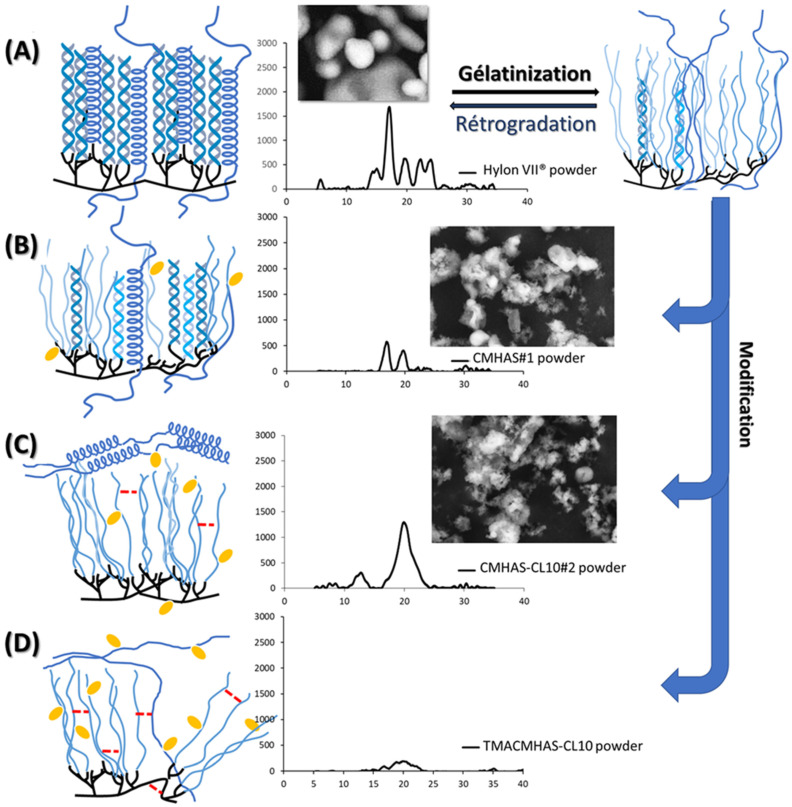
Schematic representation of the relationship between the structural organisation of starch after modifications and the X-ray diffraction pattern of (**A**) native high-amylose starch (HAS), (**B**) CMHAS, (**C**) CMHAS-CL and (**D**) ampholytic starch (TMACMHAS-CL10).

Highly viscous potato starch required dilution and hence longer evaporation time for similar film thickness, and could not be of interest for large scale film production. In addition, potato starch gelatinizes faster, forming soluble films. Corn starch generated homogeneous films, but were disintegrated in SGF. Amylose-rich starches were reported to form better films due to their capacity to orient in any direction (amylose chains shown in Figure 5 as disorganized coils, non-helical structures), in comparison to amylopectin which is too bulky to change its chain orientation and has a favored structural orientation toward small chains [61]. Due to physical (gelatinization Figure 5A) and chemical (grafting of CM anionic groups Figure 5B, crosslinking Figure 5C) modifications, the organization at molecular level is changing significantly. The films obtained from these powders had low-solubility behavior—a feature unique to HAS excipients. 

**Figure 6:**
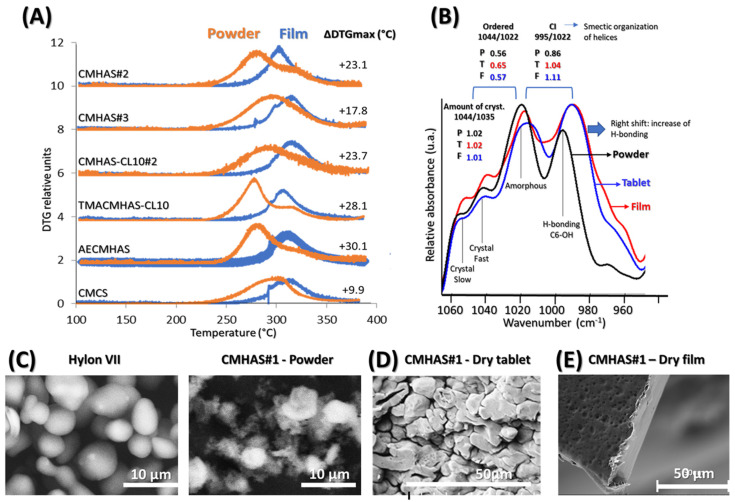
(**A**) DTG of the powders (orange) and of the films (blue) obtained from the TGA curves (10 °C/min). (**B**) Different structural parameters detailed in the ATR-FTIR deconvoluted spectra of AECMHAS in powder, tablet and film form. SEM of (**C**) starch powders, (**D**) CMHAS#1 tablet and (**E**) CMHAS#1 film.

The structural differences between the powder and film forms were evident using TGA (Figure 6A). The film, being bulkier, showed higher thermal stability than the powders. Changes were also observed in FTIR (Figure 6B), the tablet and film showing stronger bands associated to H-bonding and disorganization. SEM analysis revealed that native starch Hylon VII^®^ particles are smooth and mostly round (Figure 6C). Contrarily, all prepared derivatives showed unregular forms with thin and pale hollow around. The starches modified in aqueous phase undergo a deep modification of granules, the structure of starch particles being almost completely disorganized. This disorganized, amorphous structure explains also the low densities obtained (Table 2). For CMHAS#1 with short gelatinization times, the granular shapes were still partly present. In the tablets, compaction increases the contact between chains and also distorts the crystals. In presence of moisture, water acts as a plasticizer, generating denser and harder tablets that hydrate slowly (Figure 6D and Figure S4). In the films, the material is denser (Figure 6E), forming more H-bonds, but greatly disorganized because the retrogradation is prevented by the entanglement of the chains.

**Figure 7:**
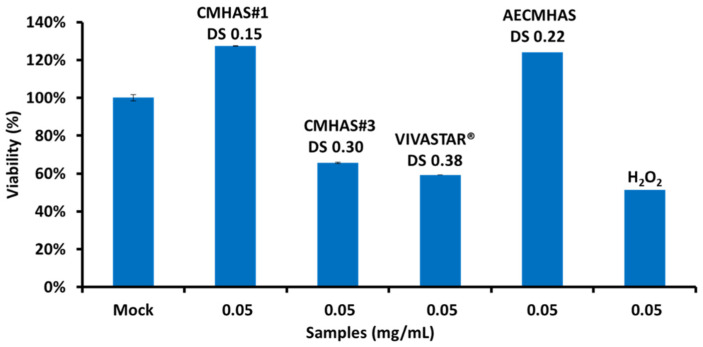
Cytotoxicity of ionic (CMHAS#1, CMHAS#3, Vivastar^®^) and ampholytic (AECMHAS) starch derivatives all at a final concentration of 0.05 mg/mL. H_2_O_2_ was used as a positive control. Untreated cells (Mock) were set at the 100% viability value.

Cytotoxicity of starch derivatives. CMHAS at a degree of substitution DS 0.15 showed no loss in cell viability (Figure 7), whereas moderate cytotoxicity was measured for CMHAS at higher substitution degree (DS 0.30). Higher toxicity of CMHAS at a higher DS could be related to the ionic disturbance of membrane proteins. Ampholytic AECMHAS derivatives demonstrated no cytotoxicity, potentially due to the compensation of carboxylic groups with the amine groups of the starch derivative. H_2_O_2_ (positive control) was cytotoxic at equivalent concentrations, as expected. For CMHAS (DS 0.15) no toxicity was found, even at higher concentrations (0.1 mg/mL).

## 4. Discussion

Correlations between structure and properties of analyzed excipients was examined in powders, tablets and films. Powders with minor chemical modifications all showed a residual crystallinity. Due to the precipitation method with acetone or alcohol that are slowly reducing the water content by dehydration, the hydroxyl groups are water-free.

They can be involved in intra-chain (helices) or inter-chain (network) hydrogen bonding with neighboring hydroxyl, carboxyl, amine or ammonium groups that will induce the starch chains to reorganize forming helices or hydrogels. In the film form (after water evaporation during drying), the amylose remained in the network, reducing the V-type crystallinity (XRD) and increasing the stability to thermal degradation (TGA) due to new starch-starch hydrogen bridges created during the casting process (Figure 6A). The powders and films from CMHAS#2 and TMACMHAS-CL10 were less stable to thermal degradation and generated soluble tablets and films. For CMCS, peaks for powder and film are more similar in comparison to those of other samples. AS opposed to HAS derivatives, the casting of corn starch derivatives did not give stable films because they do not contain sufficient amylose level to form a strong network.

Films had less V-type crystallinity than powders (as shown by XRD), whereas B-type peaks remained unchanged. If the amylose is reorganized outside the amylopectin, its crystallinity could be changed by compression of the crystal size. The CMCS powder showing a V-type crystallinity was completely amorphous once cast as films.

FTIR—Tablets and films presented a larger band than the powders at 995 cm^−1^ resulting in increased CI after tableting or processing into films (Figure 6B). This higher CI is surprising, since the XRD gave lower signal intensity. It is possible that the FTIR structural parameter CI mainly refers to the proximity of the starch chains (short-range order), increased in the tablet and film forms. Apart from Vivastar^®^, tested films revealed higher CI than powders, a similar trend was also observed for the tablets. The increasing short-range order observed in films compared to powders was induced by the hydration during the casting, which allowed reorganization into crystallites. It may also be related to the sample physical form, the film being bulkier than the powder.

The AOC did not markedly differ between powders, tablets and films from the same material, but differed between various materials. This parameter is more representative of the stability related to the retrogradation extent which depends on chemical modifications, moisture and time elapsed after synthesis.

The DTGmax of the powders was a good parameter to predict the solubility of the corresponding tablets and films, but only in the frame of similar starch sources (Tables S7 and S8). DTGmax may provide a better estimation than the% RC or% V-type obtained from XRD. The DTG_max_ also seemed more representative than the FTIR ratios to anticipate the solubility, because the FTIR values are sensitive to moisture and retrogradation (i.e., storage time after modification).

**The starch types** were determinant for the solubility of their powders, tablets and films. They also had a role in the viscosity of the filmogenic solutions. The modification of HAS required longer gelatinization time than for potato or corn starch, but generated filmogenic solutions with low viscosity and films with low solubility. The films of HAS derivatives were also more resistant during hydration compared to those of CMCS that were broken in pieces or to those based on modified potato starch that were too fragile.

Amylose-rich starches were reported to form better films due to the amylose’s capacity to orient in any direction, in comparison to amylopectin that is too bulky to change its chain orientation and has a favored orientation for the small chains in its structure [61]. This low-solubility behavior is unique to HAS. It was tuned by addition of anionic and cationic functional groups, resulting in film-forming excipients with various solubility.

The solubility of the films was increased by various chemical modifications. By increasing ionicity and reducing crystallinity, starch modifications increased the solubility of the powders, tablets and films. The gelatinization time before the reaction influenced the crystallinity and was a more important parameter for modification of HAS than for other starches. A longer gelatinization time improved the gelatinization, and the powder was soluble in cold water.

## 5. Conclusions

The study shows that the synthesis in aqueous phase consists in the complete disorganization of the starch granules and generating powders with low density and unique film- and matrix-forming features. The process is safe, affordable, green and could be further exploited in pharmaceutical industry.

The HAS is less crystalline and less soluble than other starches and appropriate derivatization may improve its tableting, gel-forming or filmogenic capacity.

Several avenues to modify starch structure were explored (crosslinking, grafting of ionic groups, DS) and their impact on the functionality of the obtained materials was investigated. As novelty, for certain starch excipients the correlations between characteristic features under powder, tablet, and film form may be the key predictive parameters that could facilitate the choice for a specific application. Thus, if the grafting of ionic groups (CM) increased the gel-forming capacity of the materials, the cross-linking increased the film strength. The DS 0.2 generated insoluble films that were degraded only in presence of α-amylase. The gel formation was clearly increased for ampholytic HAS, comparable to that of potato and corn starch derivatives.

Cross-linked carboxymethyl high-amylose starch could be considered as a multifunctional excipient for tablets and films with the added feature of tunable solubility of relevance in the gastric and enteric media.

The relationship between the parameters of the powders for all starches (Table S6A) and HAS derivatives (Table S6B) showed that BV is useful only when the starch source is the same.

From the correlation established between the different powder, film or tablet parameters (Table S7) it was found that the “gelatinization time” (GT) is related with DTG_MAX_, with the relative crystallinity (%RC) and with the amylose crystallinity (%V).

In summary, key parameters to modulate functionality of derivatives are starch source, thermal treatment, and the presence of ionic groups.

As perspective, the tunable solubility of the starch films could be a great feature to various formulations for functional coatings based on natural polymers.

## Figures and Tables

**Table 2:** Characterization of the powdered starch excipients using various methods.

	XRD		FTIR			TGA		BV		Density	DS	Viscosity
	%V- Type	%RC	AOC	Order	CI	DTG_max_ (°C)	T_w60_ (°C)	A_640_ (au)	Ratio A_525_/A_640_	(g/mL)	CM	mPa·s
**Hylon VII^®^**	23	15.4	1.08	0.70	1.20	326	321	0.78	1.34	0.50	-	1
**Gelatinized HAS**	31	9.4	1.14	0.74	1.26	326	319	0.81	1.35	0.34	-	1
**HAS-CL20**	53	9.3	0.95	0.69	1.04	313	307	0.76	1.36	0.24	-	7
**CMHAS#1**	38	5.2	0.92	0.63	1.06	307	299	0.80	1.41	0.34	0.15	1
**CMHAS#2**	100	12.4	0.84	0.56	0.88	278	281	0.31	1.40	0.27	0.34	69
**CMHAS#3**	44	4.8	1.04	0.67	1.02	293	286	0.78	1.62	0.14	0.19	16
**CMHAS-CL10#2**	75	11.4	0.85	0.57	0.96	284	286	0.68	1.47	0.16	0.19	91
**AECMHAS**	88	9.4	0.95	0.56	0.87	275	278	0.70	1.63	0.12	0.22	24
**TMACMHAS-CL10**	32	1.9	0.91	0.63	0.93	274	274	0.09	0.93	0.37	0.35	8
**PenPure^®^** (native potato starch)	10	20.4	1.13	0.77	1.24	326	320	0.41	1.18	0.75	-	1
**Vivastar^®^** (CM and CL potato starch)	5	10.4	1.12	0.80	1.23	299	300	0.03	0.63	0.80	0.38	11,010 *
**CMPS-CL10**	-	-	0.91	0.56	1.07	314	322	0.10	0.95	-	0.22	314
**Melojel^®^**	7	24.2	1.36	0.77	1.20	333	327	0.42	1.21	0.56	-	1
**CMCS**	73	9.1	0.88	0.59	0.97	301	290	0.39	1.49	0.23	0.15	70
**CMCS-CL10**	-	-	0.93	0.59	1.07	311	319	0.37	1.39	-	0.20	298

* viscosity at 1% for Vivastar.

**Table 3:** Testing and observations of the tablets. The powders were conditioned at ambient environment prior to compression (about 10% *w*/*v* moisture). Tablets (300 mg, 9.54 mm diameter) were compressed at 2 T for 10 s. The water uptake was followed at 37 °C, 100 rpm and tablet behavior was observed in the USP apparatus #2.

	Tablet	Swelling Behavior in SGF/SIF	Weight after Immersion in SGF/SIF with Pancreatic Enzymes after 7 h	Disintegration in SGF/SIF with Enzymes, 37 °C		
Excipient				Time (±15 min)	Final Residue Weight after 9 h, Dried	Erosion Rate ER (%/h)
Hylon VII^®^		--	-	-	-	
Gelatinized HAS		Capped, fractured	89%	>9 h	61%	4.3
HAS-CL20		Hydrated and cracked	135%	>9 h	78%	2.4
CMHAS#1		Hydrated and cracked	213%	>9 h	69%	3.4
CMHAS#2		Outer gel layer	740%	4 h 30	0%	22.2
CMHAS#3		Outer gel layer	270%	7 h	0%	14.3
CMHAS-CL10#1		Hydrated and cracked	65%	>9 h	69%	3.4
CMHAS-CL10#2		Outer thin gel layer	386%	>9 h	gel	11.1
AECMHAS		Outer thick gel layer	557%	7 h	gel	14.3
TMACMHAS-CL10		Outer thin gel layer	0%	3 h 30	0%	28.6
CMCS		Outer gel layer	458%	>9 h	gel	11.1
CMCS-CL10		Outer gel layer	357%	6 h	gel	16.7
CMPS-CL10		Outer gel layer	778%	6 h	gel	16.7

**Table 4:** Film related properties: Viscosity of filmogenic solutions (3.5% (*w*/*v*) of starch derivatives; Solubility of the films in SGF and SIF media; Change of the films weight during conditioning at 40%, 60% and 75% Relative Humidity (RH).

Derivative Used for Film Formulation	Viscosity	Solubility of Films (Weight Loss as% *w*/*w*) after 2 h in SGF or in SIF Followed by Drying at 60 °C for 72 h		Weight Change after Conditioning Reported to 40% RH (% *w*/*w*)		
	(mPa·s ± 5 mPa·s)	2 h SGF	2 h SIF	40% RH	60% RH	75% RH
HAS-CL20	159	1.7 ± 0.7	4.4 ± 0.4	-	-	-
CMHAS#1	29	2.6 ± 1.7	5.0 ± 1.1	0	2.0	4.8
CMHAS#2	31	100%		0	0.9	3.8
CMHAS#3	41	0 ± 0.4	4.9 ± 0.9	0	1.1	4.2
CMHAS-CL10#2	86	Fragmented	Fragmented	0	1.0	4.5
AECMHAS	52	14.0 ± 2.1	100%	0	0	Sticky
TMACMHAS-CL10	19	100%		Sticky	Sticky	Sticky
CMCS (corn starch)	107	Fragmented	10.4 ± 16.1	0	1.0	3.7

## Data Availability

Not applicable.

## Supplementary Materials

The following supporting information can be downloaded at: https://www.mdpi.com/article/10.3390/pharmaceutics15030834/s1, Figure S1: Synthesis of starch derivatives using physical and chemical modifications; Figure S2: ATR-FTIR spectra of the starch derivatives; Figure S3: FTIR structural region of the powder, tablet and film form of a few samples after correction with baseline and normalization, for calculation of the FTIR ratios; Figure S4: Up: CMHAS tablets obtained from moisturized CMHAS (left) and dry CMHAS (right). Down: the same tablets after hydration in water 15 min. Table S1: Literature and experimental values for ATR-FTIR short-range structural parameters of reference starch samples. Powders were not dried or hydrated; Table S2: FTIR and DRX parameters for powders (P), tablets (T) and films (F); Table S3: TGA and DTG values of the powder samples; Table S4: Spectrophotometric measurements of the starch-iodine complex formation for the different derivatives and native starches; Table S5: Analysis of the films using TGA. W_final_ is the weight at 350 °C; Table S6: Comparison of the correlation of the parameters for all the powder starch derivatives (A) and only for HAS (B); Tables S7 and S8: Correlation of the parameters measured for powders (P), tablets (T) and films (F).

## Abbreviations

AAC: apparent amylose content; AE: aminoethyl; AOC: amount of crystallinity; BV: blue value; CEA: chloroethylamine; CI: crystallinity index; CM: carboxymethyl; CMHAS: carboxymethyl high amylose starch; CL: cross-linked; CMHAS-CL: carboxymethyl high amylose starch cross-linked; CMCS: carboxymethyl corn starch; CMPS: carboxymethyl potato starch; DR: disintegration rate; DS: degree of substitution; DT: disintegration time; DTG: differential thermogravimetry; FTIR: Fourier-transformed infrared; GTMAC: glycidyl trimethylammonium chloride; HAS: high-amylose starch; RC: relative crystallinity; SGF: simulated gastric fluid; SIF: simulated intestinal fluid; SMCA: sodium monochloroacetate; STMP: sodium trimetaphosphate; TGA: thermogravimetric analysis; TMA: trimethylammonium; USP: United States Pharmacopeia; XRD: X-ray diffraction.
