# Supplementary material for: Anionic and Ampholytic High-Amylose Starch Derivatives as Excipients for Pharmaceutical and Biopharmaceutical Applications: Structure-Properties Correlations
Source: Pharmaceutics. 2023 Mar 3;15(3):834. doi: 10.3390/pharmaceutics15030834 (PMC10054713; doi:10.3390/pharmaceutics15030834)
Supplement: Supplementary file 1 [file pharmaceutics-15-00834-s001.zip › pharmaceutics-2152576-supplementary.pdf]

SUPPLEMENTARY MATERIALS

Anionic and Ampholytic High-Amylose Starch Derivatives as Excipients for Pharmaceutical and Biopharmaceutical Applications: Structure-Properties Correlations

Marc-André Labelle [1], Pompilia Ispas-Szabo [1,*] Salma Tajer[1], Yong Xiao[2], Benoît Barbeau[2] and Mircea Alexandru Mateescu [1]

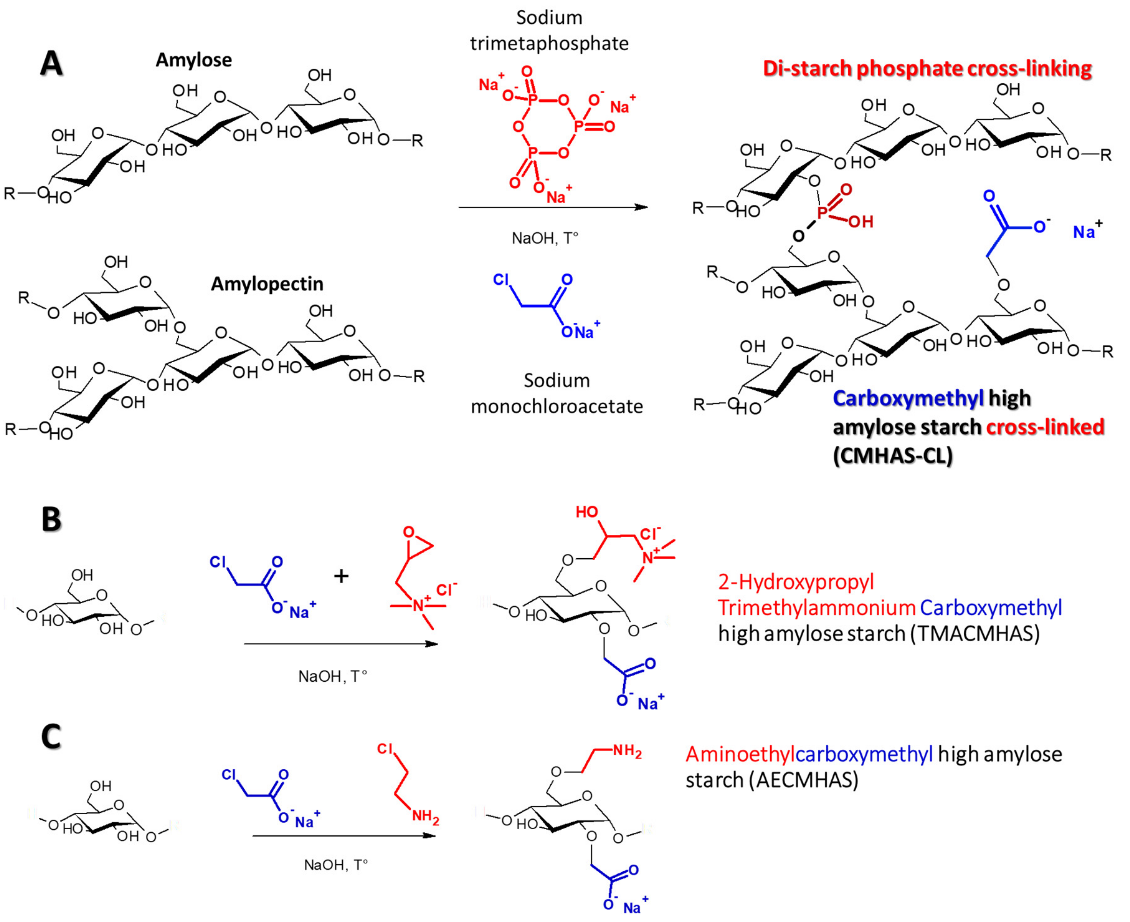

Figure S1: Synthesis of starch derivatives using physical and chemical modifications.
A) CMHAS-CL obtained by reacting starch with sodium trimetaphosphate (STMP) and sodium monochloroacetate (SMCA); B) TMACMHAS was obtained by reacting starch with SMCA and glycidyl trimethylammonium chloride; C) AECMHAS was obtained by reacting with chloroethylamine and SMCA.

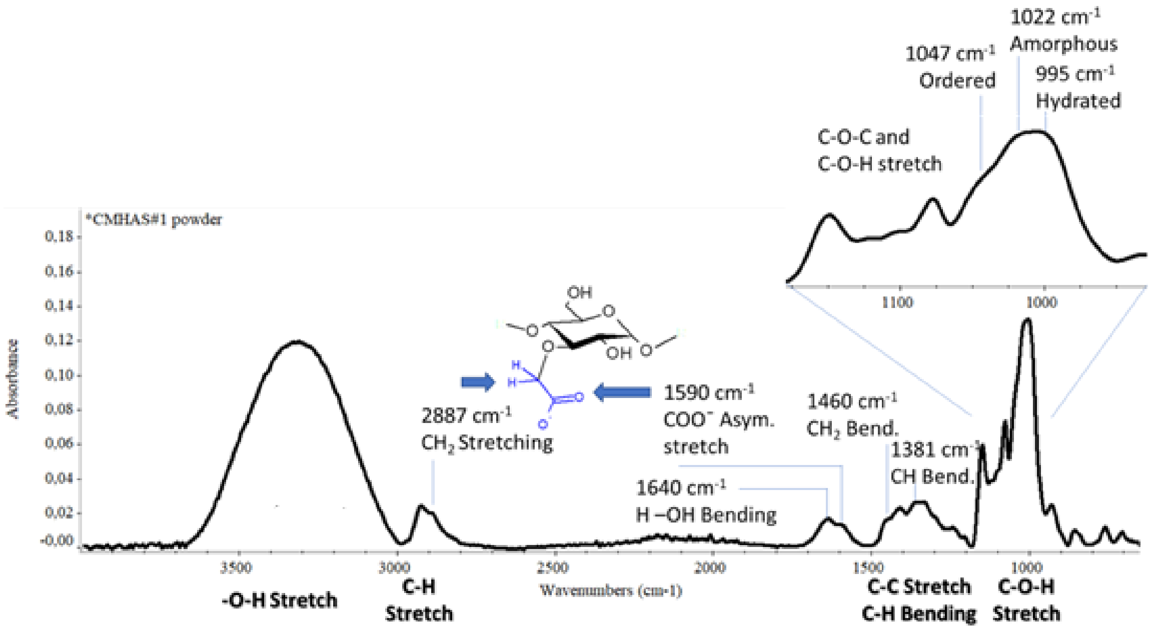

Figure S2 – Upon grafting of carboxymethyl functions, new bands appear for CH_2 and COO^- in the ATR-FTIR spectra of the derivatives. The 1200-1000 cm^{-1} region contains structural information about the short-range organization. The region 800-1185 cm^{-1} (insert) was corrected with a baseline, deconvoluted and normalized to analyze the bands 995, 1022, 1035 and 1044 cm^{-1}.

Table S1 – Literature and experimental values for ATR-FTIR short-range structural parameters of reference starch samples. Powders were not treated (dried or hydrated).

	Experimental			Others			
Starch materials	AOC 1044/ 1035	Ordered 1044/ 1022	CI 995/ 1022	AOC 1044/ 1035	Ordered 1044/ 1022	CI 995/ 1022	Reference
Wheat	-	-	-	-	0.59	-	[26]
Corn	1.36	0.77	1.20	1.1	0.61	-	[27]
Potato	1.13	0.77	1.24	-	0.79	-	[26]
HAS	1.08	0.70	1.20	-	0.75	1.03	[28]
Other starches	-	-	-	-	0.5-0.6	1.2-1.3	[29]
	-	-	-	-	0.65	-	[26]

Table S2 – FTIR and DRX parameters for powders (P), tablets (T) and films (F). The percentage of relative crystallinity (%RC) was obtained by using the background function in the software Diffract Suite. To estimate the baseline, the background parameter 'enhanced' was activated and the curvature was increased until obtaining a good fitting with the spectral baseline (*i.e.* 25%). The %V is the percentage of V-type peaks in the crystalline region.

Sample	Form	Small-range organization - FTIR			Long-range organization XRD	
		AOC 1044/1035	Ordered 1044/ 1022	C.I. 995/1022	RC (%)	V-type (%)
Hylon VII	**P**	1.08	0.70	1.20	15.4	23
Gelatinized HAS	**P**	1.14	0.74	1.26	9.4	31
	**T**	Not done			5.4	11
CMHAS#1	**P**	0.92	0.63	1.06	5.2	38
	**T**	1.02	0.73	1.07	10.6	0
	**F**	0.96	0.79	1.20	Too rigid	
CMHAS#2	**P**	0.84	0.56	0.88	12.4	100
	**T**	0.93	0.61	1.05	7.4	100
	**F**	0.96	0.63	1.06	2.2	~0
CMHAS#3	**P**	1.04	0.67	1.02	4.8	44
	**T**	1.03	0.71	1.07	3.6	50
	**F**	0.96	0.81	1.20	19.1	16
CMHAS-CL10#2	**P**	0.85	0.57	0.96	11.4	74
	**T**	0.95	0.62	1.04	7.7	70
	**F**	0.98	0.65	1.07	3.1	49
AECMHAS	**P**	0.95	0.56	0.87	9.4	88
	**T**	0.92	0.63	1.06	2.5	0
	**F**	0.97	0.57	1.11	Not done	Not done
TMACMHAS-CL10	**P**	0.91	0.63	0.93	1.9	31
	**T**	0.90	0.70	1.15	1.7	15
	**F**	0.89	0.70	1.02	0.7	0
HAS-CL20	**P**	0.95	0.69	1.04	9.3	53
	**T**	1.03	0.71	1.13	5.5	39
	**F**	0.94	0.77	1.18	Too rigid	
Corn starch Melojel (CS) ®	**P**	1.36	0.77	1.20	24.2	29
Starch1500®	**P**	0.98	0.62	1.01	10.2	26
	**T**	0.93	0.63	1.03	7.4	32
CMCS	**P**	0.88	0.59	0.97	9.1	7
	**T**	0.95	0.66	1.02	10.1	100
	**F**	1.00	0.65	1.11	2.2	37
CMCS-CL10	**P**	0.93	0.59	1.07	Not done	
	**T**	0.96	0.69	1.04		
Potato starch (PS) PenPure®	**P**	1.13	0.77	1.24	20.4	11
Vivastar®	**P**	1.12	0.80	1.23	10.4	5
	**T**	1.16	0.81	1.09	12.4	0
	**F**	0.94	0.64	1.07	3.1	0
CMPS-CL10	**P**	0.91	0.56	1.07	Not done	
	**T**	0.90	0.67	1.11		

Table S3 – TGA and DTG values of the powder samples. The water content was calculated at 150 °C; the onset was measured as the crossing of the maximum slope with the initial slope; The final weigh was the remaining mass at 350 °C; T_{W60} and T_{W50} represent the temperature at which the weigh was 50 or 60% (w/w).

**Starch materials**	**Water (%)**	**Onset (°C)**	**Final weight (%)**	**T_{w60} (°C)**	**T_{w50} (°C)**	**DTG peak (°C)**	**DTG shoulder (°C)**
**Hylon VII®**	10.0	310.8	15.1	321.0	324.6	326.3	297.7
**Gelatinized HAS**	9.7	297.9	22.5	319.1	326.1	325.8	-
**HAS-CL20**	10.3	288.5	26.5	306.8	312.5	313.1	-
**CMHAS#1**	8.9	268.7	25.5	299.2	308.8	307.2	-
**CMHAS#2**	10.4	254.3	21.8	280.7	290.5	277.9	319.4
**CMHA#3**	9.2	257.4	17.7	286.0	295.1	292.5	-
**CMHAS-CL10#2**	10.1	254.8	25.1	285.9	296.9	284.3	-
**AECMHAS**	9.9	258.3	18.3	277.9	285.7	275.3	318.8
**TMACMHAS-CL10**	11.3	255.5	19.1	273.7	280.3	274.5	314.3
**PenPure®**	12.7	308.1	22.3	320.0	324.2	325.6	298.4
**Vivastar®**	10.8	287.1	37.9	299.9	306.3	299.3	303.2
**CMPS-CL10**	9.6	288.8	46.3	321.8	**338.9**	313.9	-
**Melojel®**	10.6	313.8	22.1	327.3	331.5	332.7	301.6
**Starch1500®**	8.9	310.5	30.4	326.5	331.5	328.2	304.3
**CMCS**	10.2	261.3	17.4	289.6	298.5	301.4	-
**CMCS-CL10**	8.2	292.1	41.1	319.2	330.4	311.2	-

Table S4 – Spectrophotometric measurements of the starch-iodine complex formation for the different derivatives and native starches. The absorbance at 640 nm (A_{640}) is called the blue value (BV). Using pure amylose and pure amylopectin, a linear regression was obtained for A_{640} and also for the ratio of absorbance A_{640}/A_{525}. The error is the mean of the standard deviation of triplicates. AAC : apparent amylose content, calculated by linear regression.

	**A_{640}**	**Ratio**	**Regression**	
**Native starches**	**Blue value ± 0.02 (AU)**	**A_{640}/A_{525} ± 2 (%)**	**AAC ± 2 (%)**	**A_{640}/A_{525} ± 2 (%)**
**Amylose (AM)**	0.93	1.54	100	100
**Amylopectine (AP)**	0.33	0.86	0	0
**Hylon VII®**	0.78	1.34	74	71
**Hylon V®**	0.67	1.28	56	62
**MelojeI®**	0.42	1.21	14	52
**PenPure60®**	0.41	1.54	13	47
**Derivatives**	**Blue value**	**A_{640}/A_{525}**	**AAC**	**A_{640}/A_{525}**
**HAS gelatinized**	0.81	1.35	79	72
**HAS-CL20**	0.76	1.36	71	73
**CMHAS#1 DS0.15**	0.80	1.41	78	81
**CMHAS#2 DS0.3**	0.31	1.40	-4	80
**CMHAS#3 DS0.15**	0.78	1.62	75	111
**CMHAS-CL10#1**	0.61	1.47	46	36
**CMHAS-CL10#2**	0.68	1.63	58	89
**AECMHAS**	0.70	0.93	61	113
**TMACMHAS-CL10**	0.09	1.49	-41	**10**
**CMCS**	0.39	1.39	10	92
**CMCS-CL10**	0.37	0.95	7	78
**CMPS-CL10**	0.10	1.18	-39	**13**
**Vivastar®**	0.03	0.63	-51	-35

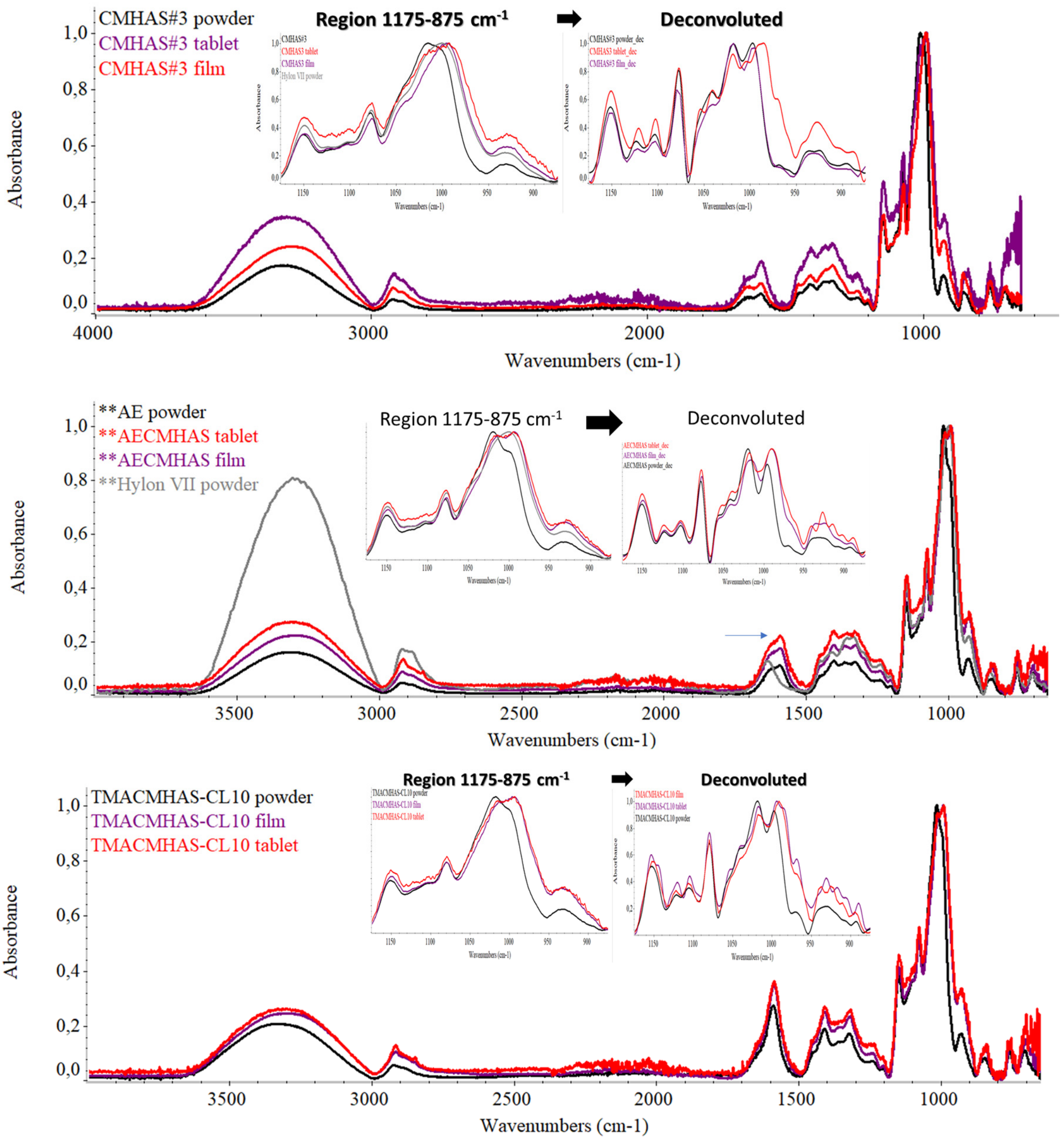

Figure S3– FTIR structural region of the powder, tablet and film form of a few samples after correction with baseline and normalization, for calculation of the FTIR ratios. In the insert: the region 1175-875 cm^{-1} before and after deconvolution.

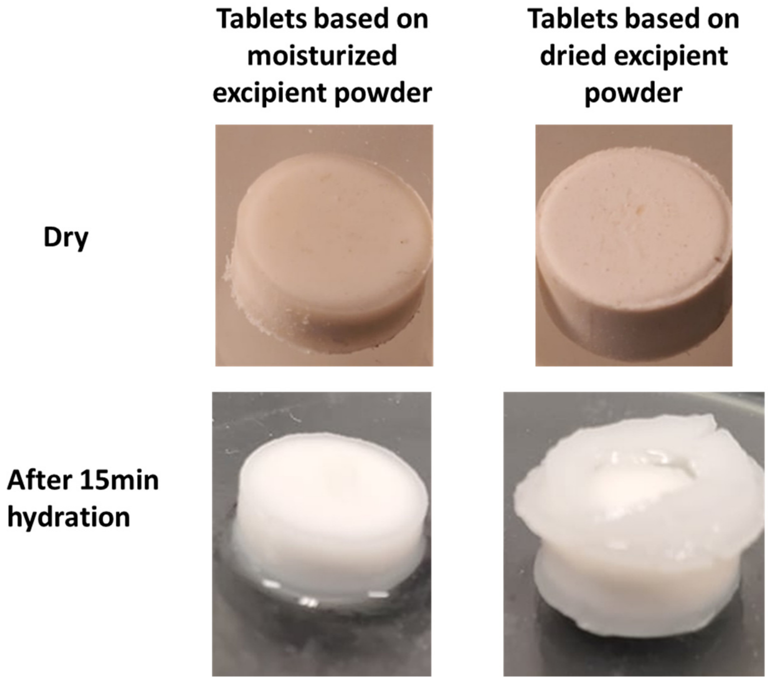

Figure S4 – Up: CMHAS tablets obtained from moisturized CMHAS (left) and dry CMHAS (right) powders. Down: the same tablets after hydration in water 15 min. The water may act as a plasticizer and impact the structural changes during the compaction process.

Table S5 - Analysis of the films using TGA method. W_{final} is the weight at 350 °C.

Starch films	Moisture (%)	W_{final} (%)	Onset (°C)	DTG peak (°C)	DTG shoulder (°C)
CMHAS#2	8.4	42.2	284.1	301.0	293.6
CMHAS#3	8.3	41.5	286.0	310.3	294.0<br>275.6
CMHAS-CL10#2	7.9	42.2	286.9	308.0	-
AECMHAS	8.0	42.7	284.8	305.3	290.9
TMACMHAS-CL10	8.8	43.5	284.3	302.6	290.4
CMCS	8.7	39.8	286.5	311.3	292.6

Table S6 - Comparison of the correlation of the parameters for all the powder starch derivatives (A) and only for HAS (B). Clear blue = good correlation, Red = no correlation. GT: gelatinization time. Tw50, Tw60, DTG and onset are TGA parameters. %RC and %V are XRD parameters. AOC, order and CI are FTIR parameters.

A)

		TGA				XRD		FTIR					
	GT	*Ons*	*T_{w50}*	*T_{w60}*	*DTG*	*%RC*	*%V*	*AOC*	*order*	*CI*	*BV*	*DS*	*Visco.*
**GT**	1.0												
**Onset**	-0.8	1.0											
**T_{w50}**	-0.8	0.9	1.0										
**T_{w60}**	-0.8	0.9	1.0	1.0									
**DTG**	-0.9	0.9	0.9	1.0	1.0								
**%RC**	-0.7	0.7	0.7	0.7	0.6	1.0							
**%V**	0.6	-0.7	-0.7	-0.7	-0.7	-0.3	1.0						
**AOC**	-0.5	0.7	0.5	0.6	0.6	0.7	-0.8	1.0					
**Order**	-0.5	0.6	0.3	0.4	0.6	0.5	-0.9	0.9	1.0				
**CI**	-0.8	0.8	0.7	0.7	0.8	0.5	-0.9	0.8	0.9	1.0			
**BV**	-0.1	0.0	0.0	0.0	0.2	-0.1	0.2	0.1	0.0	0.0	1.0		
**DS**	0.8	-0.7	-0.6	-0.7	-0.8	-0.5	0.3	-0.5	-0.4	-0.5	-0.6	1.0	
**Viscosity**	0.6	0.0	-0.2	-0.1	-0.2	-0.1	-0.4	0.2	0.5	0.3	-0.7	0.6	1.0

Full correlation | Limited | Low | No correlation

B)

	GT	*Onset*	*T_{w50}*	*T_{w60}*	*DTG*	*%RC*	*%V*	*AOC*	*order*	*CI*	*BV*	*DS*	*Visco.*
**GT**	1.0												
**Onset**	-0.8	1.0											
**T_{w50}**	-0.9	0.9	1.0										
**T_{w60}**	-0.9	1.0	1.0	1.0									
**DTG**	-0.8	0.9	1.0	1.0	1.0								
**%RC**	-0.5	0.5	0.5	0.5	0.3	1.0							
**%V**	0.5	-0.6	-0.6	-0.6	-0.7	0.3	1.0						
**AOC**	-0.4	0.7	0.7	0.7	0.7	0.1	-0.7	1.0					
**Order**	-0.5	0.8	0.8	0.8	0.9	0.0	-0.8	0.9	1.0				
**CI**	-0.7	0.9	0.9	0.9	0.9	0.2	-0.8	0.8	0.9	1.0			
**BV**	-0.6	0.5	0.7	0.6	0.7	0.3	-0.2	0.5	0.4	0.6	1.0		
**DS**	0.8	-0.9	-0.9	-0.9	-0.9	-0.4	0.5	-0.7	-0.8	-0.8	-0.8	1.0	
**Viscosity**	0.5	-0.8	-0.8	-0.8	-0.9	0.3	0.9	-0.7	-0.9	-0.8	-0.7	0.8	1.0

Full correlation | Limited | Low | No correlation

GT: gelatinization time. Onset: onset of the degradation curve of TGA. Tw50 and Tw60: temperature at 50 or 60% weight (TGA). DTG: highest peak of the TGA differential curve. %RC: relative crystallinity (XRD). %V: % amylose type-V (XRD). AOC: amount of crystallinity (FTIR ratio 1044/1035), order: FTIR ratio 1044/1022. CI: crystallinity index (FTIR ratio 995/1022). BV: blue value, DS: degree of substitution of hydroxyls by carboxymethyl functions. The conclusion is that a better correlation was found when comparing the same starch type and that the XRD parameter %V was more representative than the %RC. When comparing different starches, the blue value and viscosity did not correlate.

Table S7 – Correlation of the parameters measured for powders (P), tablets (T) and films (F) for all types of modified starches.

Starches	*GT*	*PDTG*	*P%V*	*PAOC*	*Pord*	*PCI*	*PBV*	*PDS*	*Pvis*	*TDR*	*Tsw*	*Tsol*	*Taoc*	*Tord*	*TCI*	*T%V*	*Fsol*	*Faoc*	*Ford*	*FCI*	*Fons*	*FDTG*	*F%V*
GT	1.0																						
PDTG	-0.8	1.0																					
P%V	0.4	-0.6	1.0																				
PAOC	-0.2	0.5	-0.8	1.0																			
Pord	-0.3	0.4	-0.9	0.9	1.0																		
PCI	-0.7	0.7	-0.8	0.8	0.8	1.0																	
PBV	-0.3	0.3	0.1	0.2	0.1	0.1	1.0																
PDS	0.7	-0.8	0.1	-0.3	-0.2	-0.4	-0.8	1.0															
Pvis	0.5	-0.2	-0.6	0.5	0.7	0.5	-0.8	0.6	1.0														
TDR	0.7	0.3	-0.6	0.4	0.5	0.4	-0.5	0.0	0.7	1.0													
Tsw	0.5	-0.7	0.4	-0.6	-0.6	-0.6	-0.6	0.6	-0.3	-0.5	1.0												
Tsol	0.5	-0.4	0.3	-0.2	-0.4	-0.4	-0.6	0.6	0.3	0.2	0.5	1.0											
Taoc	-0.2	0.2	-0.7	0.8	0.9	0.8	0.1	0.1	0.9	0.4	-0.7	-0.3	1.0										
Tord	-0.2	0.2	-1.0	0.7	0.9	0.8	-0.2	0.2	0.8	0.3	-0.3	-0.3	0.8	1.0									
TCI	0.2	-0.1	-0.6	0.2	0.4	0.2	-0.3	0.2	0.3	-0.1	0.3	-0.3	0.1	0.5	1.0								
T%V	0.4	-0.6	0.9	-0.8	-0.8	-0.8	0.0	0.1	-0.5	-0.5	0.8	0.5	-0.7	-0.8	-0.5	1.0							
Fsol	0.5	-0.8	0.1	-0.1	-0.2	-0.2	-0.6	0.8	0.5	0.3	0.5	0.6	-0.2	-0.2	0.1	0.2	1.0						
Faoc	-0.8	0.1	0.5	-0.7	-0.7	-0.5	0.6	-0.9	-0.8	-0.8	0.3		-0.7	-0.6	-1.0	0.8	-0.8	1.0					
Ford	-0.8	0.7	-0.3	-0.1	0.3	0.4	-0.4	0.0	-0.2	0.2	-0.4		0.3	0.2	-0.3	0.2	-0.4	0.2	1.0				
FCI	-0.1	0.1	0.2	0.0	-0.3	-0.3	0.4	-0.7	-0.2	-0.3	0.1		-0.3	-0.1	-0.4	0.2	-0.6	0.6	-0.5	1.0			
Fons	-0.8	0.8	-0.1	0.0	0.1	0.7	0.6	-0.9	0.6	-0.8	-0.8		0.6	0.0	-0.6		-0.5	0.8	0.6	0.2	1.0		
FDTG	-0.6	0.9	-0.2	0.4	0.3	0.8	0.6	-0.9	0.2	-0.8	-0.8		0.7	0.3	-0.5		-0.8	1.0	0.4	0.6	0.9	1.0	
F%V	-0.9	0.2	-0.8	0.2	0.3	0.8	1.0	-0.7	0.9	-0.9	-0.9		0.9	0.2	-0.3		0.0	0.5	0.9	0.2	0.9	0.7	1.0

Full correlation | **Limited** | **Low** | **No correlation**

Table S8 – Correlation of the parameters measured for powders (P), tablets (T) and films (F) for modified high-amylose starches.

HAS	*GT*	*PDTG*	*P%V*	*PAOC*	*Pord*	*PCI*	*PBV*	*PDS*	*Pvis*	*TDR*	*Tsw*	*Tsol*	*Taoc*	*Tord*	*TCI*	*T%V*	*Fsol*	*Faoc*	*Ford*	*FCI*	*Fons*	*FDTG*	*F%V*
GT	1.0																						
PDTG	-0.8	1.0																					
P%V	0.5	-0.7	1.0																				
PAOC	-0.4	0.7	-0.7	1.0																			
Pord	-0.5	0.9	-0.8	0.9	1.0																		
PCI	-0.7	0.9	-0.8	0.8	0.9	1.0																	
PBV	-0.6	0.7	-0.2	0.5	0.4	0.6	1.0																
PDS	0.8	-0.9	0.5	-0.7	-0.8	-0.8	-0.8	1.0															
Pvis	0.5	-0.9	0.9	-0.7	-0.9	-0.8	-0.7	0.8	1.0														
TDR	0.8	-0.8	0.3	-0.4	-0.5	-0.7	-0.9	0.9	0.8	1.0													
Tsw	0.7	-0.9	0.3	-0.6	-0.6	-0.7	-0.9	0.9	0.8	1.0	1.0												
Tsol	0.8	-0.9	0.5	-0.4	-0.7	-0.7	-0.5	0.8	1.0	0.8	0.7	1.0											
Taoc	-0.5	0.9	-0.4	0.6	0.7	0.9	0.8	-0.8	-0.9	-0.8	-0.9	-0.7	1.0										
Tord	-0.3	0.7	-0.9	0.6	0.9	0.8	0.2	-0.4	-1.0	-0.4	-0.4	-0.6	0.7	1.0									
TCI	0.0	0.2	-0.7	0.2	0.6	0.2	-0.4	-0.1	-0.8	0.2	0.2	-0.3	0.0	0.6	1.0								
T%V	0.5	-0.9	0.9	-0.8	-0.7	-0.9	-0.7	0.6	0.9	0.8	0.8	0.9	-0.9	-1.0	-0.5	1.0							
Fsol	0.4	-0.9	0.6	-0.7	-0.8	-0.9	-0.6	0.7	1.0	0.7	0.8	0.7	-1.0	-0.8	-0.2	0.9	1.0						
Faoc	-0.9	0.7	-1.0	0.1	0.9	0.8	0.8	-0.9	1.0	-1.0	-1.0		0.7	0.5	-0.5			1.0					
Ford	-0.7	0.9	-0.3	-0.9	0.7	0.8	-0.3	0.1	1.0	0.0	-0.2		0.9	-0.7	-1.0			0.2	1.0				
FCI	0.3	-0.6	-0.1	1.0	-0.3	-0.4	0.7	-0.5	1.0	-0.4	-0.2		-0.6	0.9	0.8			0.2	-0.9	1.0			
Fons	-0.8	0.7	-0.1	0.2	0.2	0.7	0.7	-0.9	1.0	-0.8	-0.8		0.6	0.0	-0.5		-0.4	1.0	0.5	-0.1	1.0		
FDTG	-0.4	0.8	-0.3	0.6	0.5	0.8	0.8	-0.9	1.0	-0.8	-0.9		0.8	0.4	-0.3		-0.7	1.0	0.1	0.3	0.9	1.0	
F%V	-1.0	1.0	-1.0	1.0	1.0	1.0	1.0	-1.0	1.0	-1.0	-1.0		1.0	1.0	-1.0			1.0	1.0	1.0	1.0	1.0	1.0

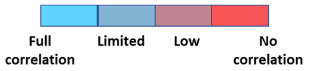

GT: gelatinization time before chemical modification, DTG: temperature at the highest peak on the DTG (TGA), %V: ratio of amylose (XRD), AOC: FTIR ratios 1044/1035, order: FTIR ratio 1044/1022, CI: FTIR ratio 995/1022. Vis: viscosity; sol: solubility, SW: swelling. Blank spaces appear when there are few data with no variation. The conclusion is that the correlation was low when comparing only HAS derivatives but when comparing different starches, the power DTG and powder viscosity were the best parameters to correlate with all the other parameters.
